# ZnO/Ag/Ag_2_WO_4_ photo-electrodes with plasmonic behavior for enhanced photoelectrochemical water oxidation

**DOI:** 10.1039/c8ra10141h

**Published:** 2019-03-12

**Authors:** Rania E. Adam, Mahsa Pirhashemi, Sami Elhag, Xianjie Liu, Aziz Habibi-Yangjeh, Magnus Willander, Omer Nur

**Affiliations:** Department of Sciences and Technology, Linköping University, Campus Norrköping SE-601 74 Norrköping Sweden rania.elhadi.adam@liu.se omer.nour@liu.se; Department of Chemistry, Faculty of Sciences, University of Mohaghegh Ardabili P. O. Box 179 Ardabil Iran; Department of Physics, Chemistry and Biology (IFM), Linköping University 58183 Linköping Sweden

## Abstract

Ag-based compounds are excellent co-catalyst that can enhance harvesting visible light and increase photo-generated charge carrier separation owing to its surface plasmon resonance (SPR) effect in photoelectrochemical (PEC) applications. However, the PEC performance of a ZnO/Ag/Ag_2_WO_4_ heterostructure with SPR behavior has not been fully studied so far. Here we report the preparation of a ZnO/Ag/Ag_2_WO_4_ photo-electrode with SPR behavior by a low temperature hydrothermal chemical growth method followed by a successive ionic layer adsorption and reaction (SILAR) method. The properties of the prepared samples were investigated by different characterization techniques, which confirm that Ag/Ag_2_WO_4_ was deposited on the ZnO NRs. The Ag_2_WO_4_/Ag/ZnO photo-electrode showed an enhancement in PEC performance compared to bare ZnO NRs. The observed enhancement is attributed to the red shift of the optical absorption spectrum of the Ag_2_WO_4_/Ag/ZnO to the visible region (>400 nm) and to the SPR effect of surface metallic silver (Ag^0^) particles from the Ag/Ag_2_WO_4_ that could generate electron–hole pairs under illumination of low energy visible sun light. Finally, we proposed the PEC mechanism of the Ag_2_WO_4_/Ag/ZnO photo-electrode with an energy band structure and possible electron–hole separation and transportation in the ZnO/Ag/Ag_2_WO_4_ heterostructure with SPR effect for water oxidation.

## Abstract 2

Ag-based compounds are excellent co-catalyst that can enhance harvesting visible light and increase photo-generated charge carrier separation owing to its surface plasmon resonance (SPR) effect in photoelectrochemical (PEC) applications.

## Graphical abstract

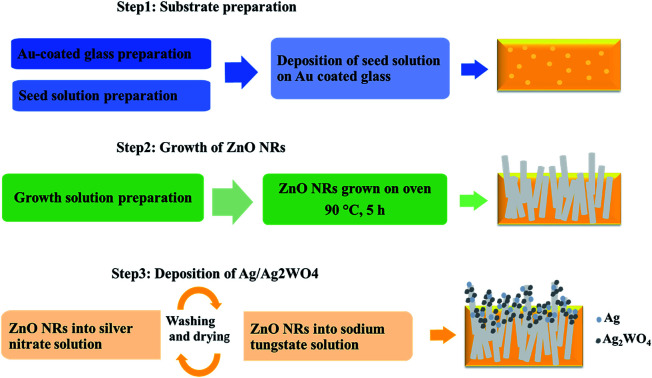

## 1 Introduction

Solar driven photocatalysis activities of semiconductors (*i.e.* dye photodegradation, hydrogen production, and CO_2_ reduction, *etc.*) have recently gained great interest because they are related to the utilization of a sustainable energy source and hence are of positive impact to the environment and energy availability issues.^1–4^ Photoelectrochemical (PEC) applications are promising for water splitting to produce hydrogen and oxygen *via* the conversion of solar energy to chemical energy.^1^ Various nanostructured metal oxides have been investigated for PEC applications such as WO_3_, TiO_2_, Fe_2_O_3_, BiVO_4_, and ZnO.^5–10^ From above mentioned semiconductors, ZnO is the most favorable due to its wide band gap (*E*_g_ ∼ 3.3 eV), and relatively high carriers mobility.^8,11–15^ ZnO possesses many point defects that form many shallow and deep levels within the bandgap resulting in deep level emission (DLE). These point defects are introduced into the crystal lattice of the ZnO nanostructures during the growth and will increase the materials photocatalytic activities within the visible light spectrum and can shift the absorption towards the visible light band from 400 nm and up to 700 nm by creating intermediates states preventing electron–hole pair recombination and enhance photocatalytic activities.^16^ These defects explain all of the visible colors of luminescence observed from different ZnO samples.^17,18^ However the high recombination rate of photo-generated charge carriers are the most influential factor that limits the efficiency of the photocatalytic processes of the ZnO.^1,12,19–21^ To tackle these obstacle, and to increase the photocatalytic activities of the ZnO under visible solar light, variety of studies are conducted to increase the photocatalytic response of the ZnO through coupling with other semiconductors or photosensitizer to form an efficient heterostructure material.^12,20,21^ Currently, Ag-based compounds are regarded as an excellent candidate as a co-catalyst that can largely enhance solar energy conversion efficiency and charge separation, which lead to further boost the PEC performance. Recent studies have proven that the deposition of Ag-containing species on the surface of composites, can lead to effectively improve harvesting visible light and increase the photo-generated charge carriers separation owing to the surface plasmon resonance (SPR) effect.^12,19,21,22^ The net result will be an enhanced PEC activity of the Ag containing composites. In this regard, silver tungsten (Ag_2_WO_4_) with a band gap between 2.9–3.1 eV, have been used for preparation of different outstanding plasmonic photo-catalysts. For example, Vignesh *et al.*^23^ studied the photocatalytic activity of Ag_2_WO_4_/g-C_3_N_4_ nanocomposite for degradation of methylene blue (MB) under solar light radiation. Their result showed an enhancement on the degradation efficiency of MB. Also, Jingjing Li^24^ investigated the formation of Ag_2_WO_4_/AgX (X = Cl, Br, I) hybrid nanorods to enhance visible light driven PEC properties. Recently, Pirhashemi *et al.*^20^ reported a highly enhanced photodegradation of organic pollutants with a plasmonic ZnO/Ag/Ag_2_WO_4_ heterostructures. Very recently, an effective PEC performance is achieved through Ag_2_WO_4_–AgX (X = Cl, Br, I) sensitized TiO_2_ nanotube array, and the deposition of the Ag_2_WO_4_ was carried out by the successive ionic layer adsorption and reaction (SILAR) method.^25^ According to the literature review, Ag/Ag_2_WO_4_ is a promising candidate to be used to develop a plasmonic sensitizer for ZnO nanostructures for optimum utilization of the solar power and accelerating charge transfer, leading to greatly enhance the PEC activities. Considering the above review, we report in this work the synthesis, characterization, and PEC activities of Ag/Ag_2_WO_4_ grown on top of ZnO nanorods (NRs). Firstly, ZnO NRs is synthesized using the hydrothermal low temperature chemical method. This was followed by the Ag/Ag_2_WO_4_ deposition on top of the ZnO NRs using the SILAR method. To the best of our knowledge there are no reports about the preparation and study of a plasmonic ZnO/Ag/Ag_2_WO_4_ photo-electrode for PEC activities. Our results showed an enhancement on the photocurrent and the current–voltage measurements. These observations are promising results for water splitting applications.

## 2 Experimental part

### 2.1 Photoelectrode preparation

**Fig. 1:**
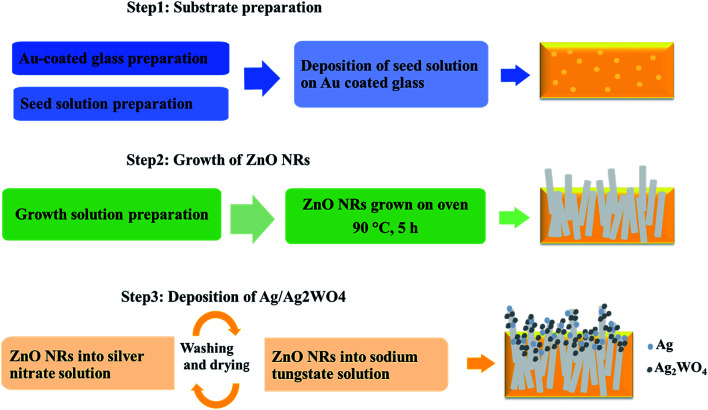
Schematic diagram of preparation of ZnO NRs and the ZnO/Ag/Ag_2_WO_4_ photoelectrodes.

The photoelectrode prepared in three steps: substrate preparation, growth of ZnO NRs, and deposition of Ag/Ag_2_WO_4_ as shown in the schematic diagram in Fig. 1 which explained in the following section.

#### 2.1.1 Substrate preparation

##### 2.1.1.1 Au coated glass preparation

In our work we have been using Au coated glass substrate that is prepared as described below, because Au coated glass has been used by many researchers as efficient electrode after deposition of ZnO based nanostructures materials,^26^ and with SPR effect.^27^ Also, it is found that the stability of electrodes can be improved by deposition of thin layer of gold.^28^ Therefore, Au coated glass was used as substrate to grow ZnO NRs and ZnO/Ag/Ag_2_WO_4_ heterostructure for PEC performance. For Au coated glass preparation, glass substrates were cleaned with acetone, isopropanol, and deionized water, respectively under ultrasonic bath for about 15 min. Then, the substrates were fixed into a vacuum chamber of an evaporator instrument. After that, an adhesive layer of 20 nm of titanium was evaporated followed by a 100 nm thickness layer of gold.

##### 2.1.1.2 Deposition of seed layer

Then a seed solution contains ZnO nanoparticles (NPs) deposited on to the substrate *via* spin coating at 3000 rpm for 25 s. The spin coating was repeated three times to have full and uniform coverage of the ZnO NPs onto the substrate. After that, the substrates were dried into an air oven at 120 °C for 10 min. The ZnO seed precursor was prepared by adding potassium hydroxide (KOH) solution (0.03 M in methanol) drop wise into zinc acetate dehydrate solution (0.01 M in methanol) under magnetic stirring (750 rpm) at 60 °C for 2 h.

#### 2.1.2 Growth of ZnO NRs

The ZnO NRs were grown on the above prepared substrates containing the seed layer of ZnO NPs by low temperature aqueous chemical growth.^29^ The precursor solution was prepared by dissolving equal molecular (0.05 M) of zinc nitrate hexahydrate (Zn (NO_3_)_2_·6H_2_O) and hexamethylenetetramine (HMT) in deionized (DI) water. The substrates that contain seed layer were immersed horizontally after they were fixed in Teflon sample holder into the precursor solution and loaded into a preheated oven at 90 °C for 5 hours. After the samples were cooled down to the room temperature, they were rinsed with DI water to remove any undesired particles, then dried with blowing nitrogen for few seconds and kept for further process.

#### 2.1.3 Deposition of Ag/Ag_2_WO_4_ on ZnO NRs

Ag/Ag_2_WO_4_ was deposit on the prepared ZnO NRs using SILAR method. An anionic and cationic aqueous precursor solutions was prepared separately using 0.05 M of silver nitrate Ag(NO)_3_ and 0.05 M of sodium tungstate (Na_2_WO_4_·2H_2_O), respectively. The deposition take place by immersion of the prepared ZnO NRs sample into Ag(NO)_3_ solution for 2 minutes to absorb the silver ions (Ag^+^) and then they were washed with DI water to remove excess ions or any other particles. Then the sample immersed into the Na_2_WO_4_·2H_2_O solution for 2 minutes and again washed with DI water. This cycle was repeated for 10 times to obtain enough Ag/Ag_2_WO_4_ particles on the ZnO NRs. Also, Ag/Ag_2_WO_4_ was deposited on ZnO NRs that grown on a pure glass substrate for some optical characterization.

### 2.2 Characterization

Powder X-ray diffraction (XRD) was used to study the structural properties of the prepared samples using Philips powder diffractometer (1729 PW) equipped with (Cu Kα) radiation with generator running at voltage of 40 kV and current of 40 mA. Field emission scanning electron microscope (FE-SEM) using a LEO 1550 Gemini field emission gun at 15 kV was used to investigate the morphology of the prepared samples. The corresponding energy depressive X-ray (EDX) with EDX mapping was investigated to identify the elemental and chemical properties of the prepared samples. The absorption spectra of the prepared samples were characterized by Perkin Elmer Lambda 900 UV-visible spectrophotometer. The chemical composition of the samples was investigated using X-ray photoelectron spectroscopy (XPS) which recorded by Scienta ESCA-200 spectrometer using monochromatic Al Kα X-ray source with a power of (1486.6 eV).

The photoelectrochemical activities were studied by using three electrode photoelectrochemical measurements using SP-200 potentiostat (Bio-Logic, Claix, France). A platinum (Pt) sheet was used as the counter electrode and a standard Ag/AgCl in 3 M KCl (as a reference electrode) was used with (0.1 M) of sodium sulfate (Na_2_SO_4_) electrolyte. The total area of the electrode that immersed in the electrolyte was 1 cm^2^. The sun light was obtained by a solar simulator that uses a 100 W ozone free xenon lamp with an output power of 1 sun (AM 1.5).

## 3 Result and discussion

### 3.1 Characterization analysis

**Fig. 2:**
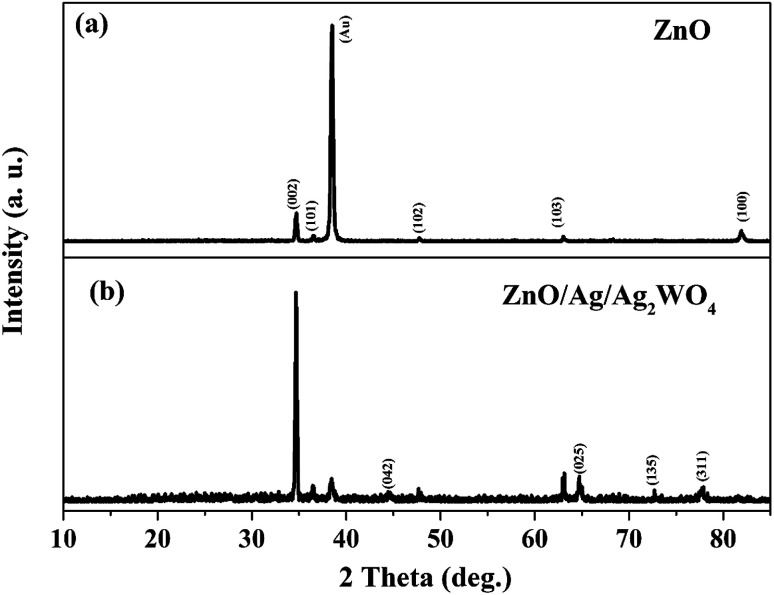
XRD patterns of the ZnO NRs and the ZnO/Ag/Ag_2_WO_4_ heterostructure.

Fig. 2 shows the structural investigation by XRD for ZnO and ZnO/Ag/Ag_2_WO_4_ samples. It could be observed that all the obtained XRD diffraction peaks in Fig. 2(a) are belonging to the hexagonal wurtzite pure phase of ZnO (JCPDS no. 36-1451) which suggest that there are no other phases of ZnO or impurities have been observed. In the XRD pattern of ZnO/Ag/Ag_2_WO_4_ heterostructure (Fig. 2(b)), more peaks were identified, which were assigned to the planes (042), (025), and (135) for Ag_2_WO_4_ (JCPDS no. 33-1195). The peak at 78° is assigned to the reflections of cubic Ag (JCPDS no. 65-2871).^20^

**Fig. 3:**
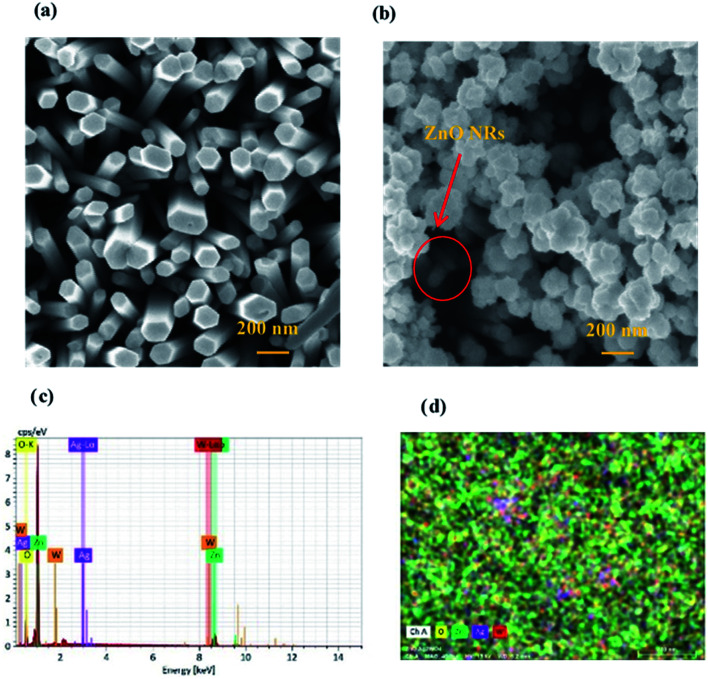
(a) and (b) FM-SEM images of the ZnO NRs and the ZnO/Ag/Ag_2_WO_4_ heterostructure. (c) EDX spectrum showing the elements composition peaks from Zn, O, Ag, and W of ZnO/Ag/Ag_2_WO_4_ heterostructure, and (d) EDX mapping show the elements distribution for the ZnO/Ag/Ag_2_WO_4_ heterostructure.

Fig. 3(a) and (b), shows the morphology of ZnO NRs and ZnO/Ag/Ag_2_WO_4_ heterostructure that was measured by the FE-SEM imaging. Fig. 3(a) shows the SEM image of the ZnO NRs, which reveal that the ZnO NRs were vertically aligned and are having hexagonal shape as expected. The diameter size of the ZnO NRs found to be ∼100 nm. After deposition of the Ag/Ag_2_WO_4_ on the ZnO NRs, a heterostructure was formed and Ag/Ag_2_WO_4_ particles were distributed on the surface of the ZnO NRs as it can be seen in Fig. 3(b). Ag/Ag_2_WO_4_ nanoparticles size were estimated from SEM imaging to vary between ∼30 to ∼150 nm, the bigger size of the nanoparticles might be due to the agglomeration of smaller nanoparticles. It is worth to note that the SPR effect depends on the size and the shape of the nanostructure, and it is quite unique in the nanostructures size from 10 to 100 s of nanometers.^30^ Therefore, the size of the prepared ZnO/Ag/Ag_2_WO_4_ heterostructure are favorable for SPR effect. The corresponding EDX spectrum of the ZnO/Ag/Ag_2_WO_4_ heterostructure were examined to show the composition of elements in the sample which consists of Zn, O, Ag, and W without any other elements detected (see Fig. 3(c)).

**Fig. 4:**
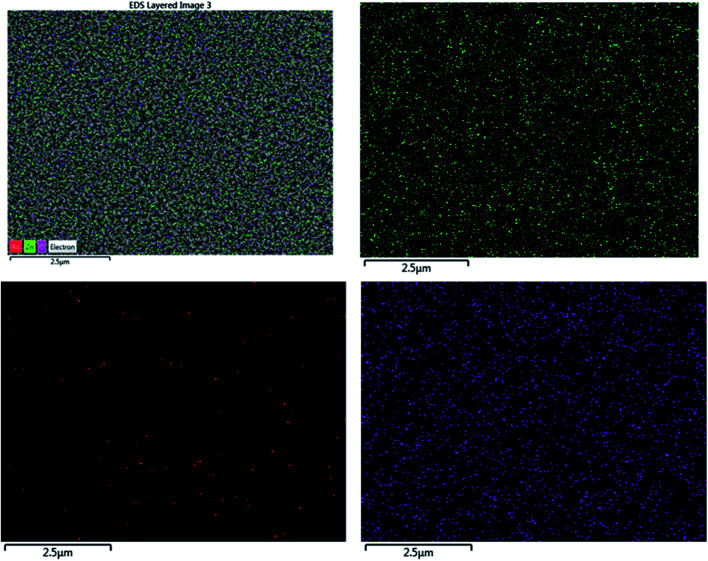
EDX mapping show the elements distribution for the ZnO NRs after immersion on the AgNO_3_ solution and before the synthesis of the Ag_2_WO_4_ for Ag nanoparticles detection confirmation. The red dots represent Ag that appears very clearly to exist on top of the ZnO NRs.

The EDX result is in good agreement with the XRD result. To further understand the distribution of the elements, the ZnO/Ag/Ag_2_WO_4_ photo-electrode was further studied by elemental mapping analysis, as shown in Fig. 3(d). From the present elementals map with particular colors for each element, it is clear that the Zn, O, Ag and W components are uniformly distributed on the sample. It is worth noting that better distribution provides strong physical coupling between counterparts. Hence, it is beneficial to efficient generation and separation of charge carriers which leads to superior PEC performance of the nanocomposite. Moreover, the EDX mapping of the ZnO NRs after it was immersed into the Ag (NO_3_) solution and before the synthesis of the Ag_2_WO_4_ was examined for the confirmation of the Ag nanoparticles existence into the heterostructure. As it can be seen in Fig. 4, Ag was detected.

**Fig. 5:**
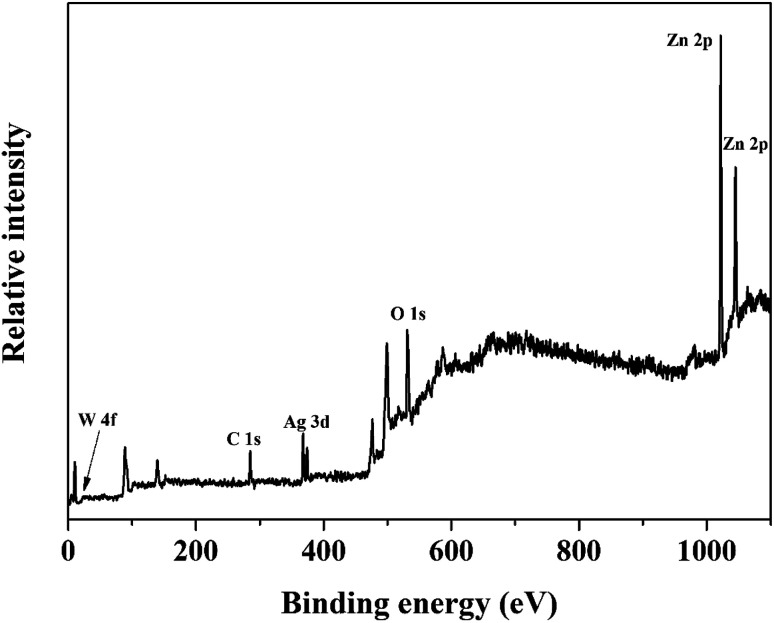
XPS spectrum survey scan of the ZnO/Ag/Ag_2_WO_4_ heterostructure.

Furthermore, the chemical state of the elements in ZnO/Ag/Ag_2_WO_4_ heterostructure were examined by XPS measurements. The XPS peaks of the all elements in the ZnO/Ag/Ag_2_WO_4_ are observed in Fig. 5. The observed XPS spectrum shown in Fig. 5 is in agreement with the EDX result that was presented in Fig. 3.

**Fig. 6:**
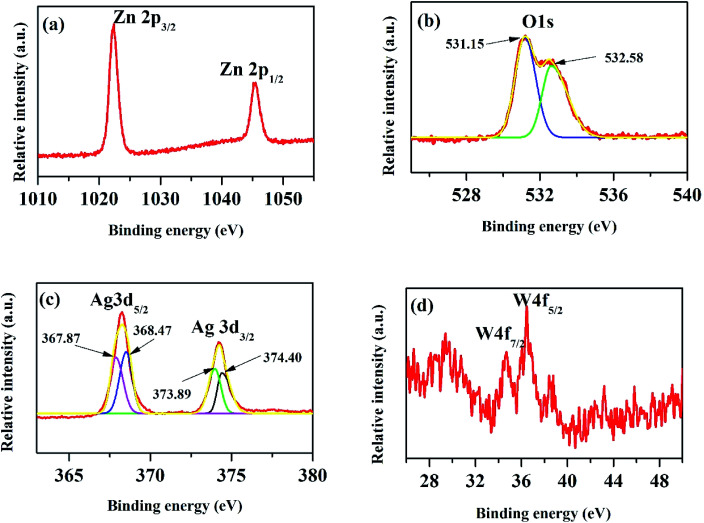
XPS core level spectra of the ZnO/Ag/Ag_2_WO_4_ heterostructure.

The XPS peak of C 1s at 284.6 eV is related to carbon from the XPS instrument. Fig. 6(a) shows the XPS core level spectra of Zn 2p of ZnO/Ag/Ag_2_WO_4_ heterostructure which is composed of two peaks centered at 1022.43 and 1045.25 eV, which are attributed to the binding energy lines of Zn 2p_3/2_ and Zn 2p_1/2_, respectively and they represented the formation of Zn–O bonds within the ZnO crystal lattice.^31,32^Fig. 6(b) shows the O1s core level XPS spectra of ZnO/Ag/Ag_2_WO_4_ heterostructure which is divided into two peaks. The peak at low binding energy centered at 531.15 eV, is related to oxygen deficient region, whereas, the peak at higher binding energy centered at 532.58 eV can be ascribed to the oxygen on the ZnO surface and water molecules H_2_O.^19,27^ The XPS spectrum of Ag 3d is shown in Fig. 6(c). The peaks at 368.21 and 374.23 eV are assigned to Ag 3d_5/2_ and Ag 3d_3/2_, respectively. The Ag 3d_5/2_ is further divided into two different peaks at 367.87 and 368.47 eV and the Ag 3d_3/2_ peak is also divided into two different peaks at 373.89 and 374.40 eV. The peaks at low energies 367.87 and 373.89 eV are accounted for the Ag^+^ in Ag_2_WO_4_, whereas, the peaks at higher energies 368.47 and 374.40 is related to metallic Ag^0^.^20,33^Fig. 6(d) shows the binding energy of W 4f which centered at 34.68 and 36.42 eV for W 4f_7/2_ and W 4f_5/2_, respectively which is consistent with those of pure Ag_2_WO_4_.^33^ The above XPS discussed results indicate the successful demonstration of ZnO/Ag/Ag_2_WO_4_ heterostructures as intended.

**Fig. 7:**
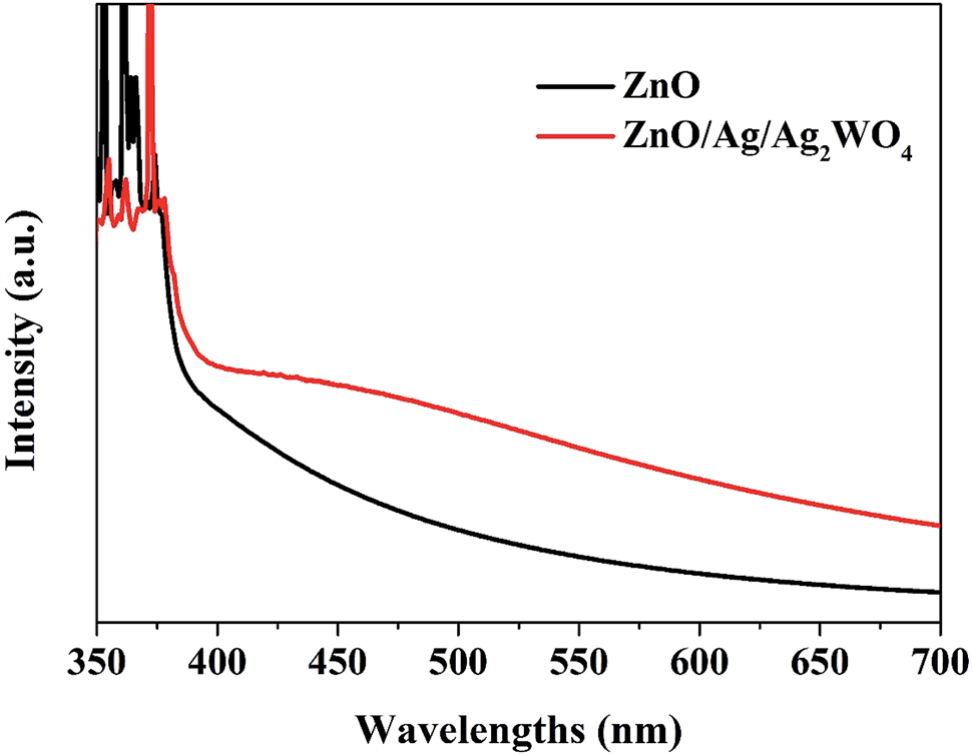
UV-vis absorption spectra of the ZnO NRs and the ZnO/Ag/Ag_2_WO_4_ heterostructure.

The UV-vis absorption spectra of the ZnO and ZnO/Ag/Ag_2_WO_4_ heterostructure show similar absorption trends (see Fig. 7). Compared to pristine ZnO NRs, ZnO/Ag/Ag_2_WO_4_ heterostructure exhibits an obvious red shift of the optical absorption in the visible region (>400 nm). The optical band gaps were found to be 3.2 and 3.1 eV for ZnO and ZnO/Ag/Ag_2_WO_4_, respectively. This result could be explained due to formation of Ag_2_WO_4_ on the top of the ZnO NRs forming the heterostructure (*i.e.* bandgap engineering). Also, note that metallic silver could be produced during the sample preparation and can trigger surface plasmonic effect.^20,21^

### 3.2 Photoelectrochemical and water oxidation analysis

**Fig. 8:**
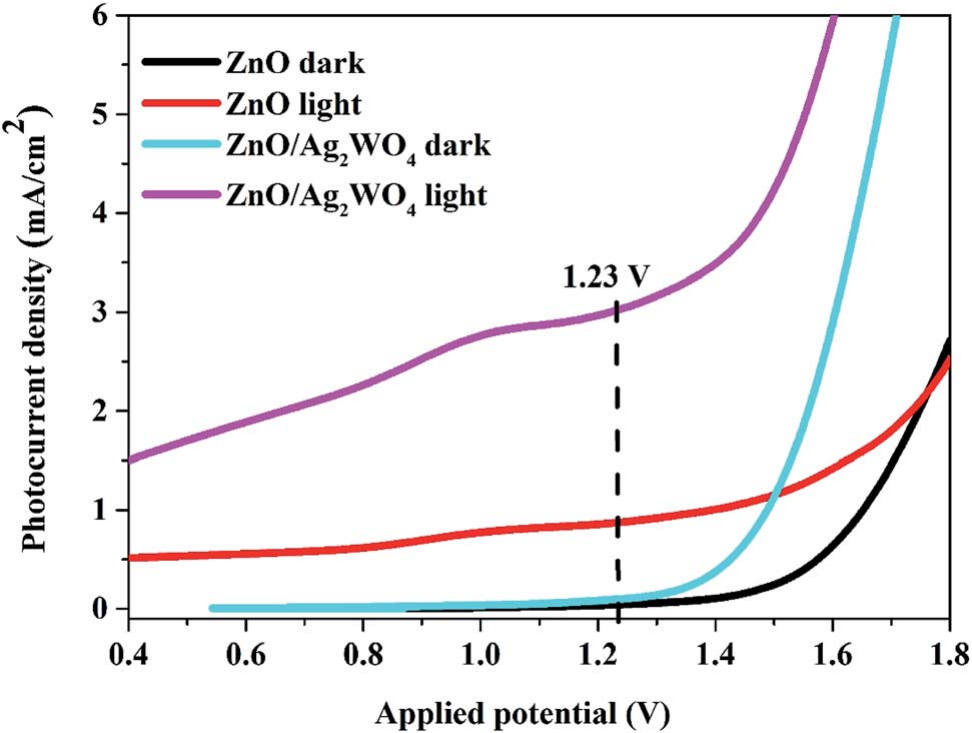
Linear sweep voltammetry curves of the ZnO NRs, and the ZnO/Ag/Ag_2_WO_4_ photo-electrodes under light and dark conditions.

The charge carrier characteristics at the semiconductor/electrolyte interface for pristine ZnO NRs and ZnO/Ag/Ag_2_WO_4_ heterostructure were examined *via* linear sweep voltammetry measurements as shown in Fig. 8. From Fig. 8 both pristine ZnO NRs and ZnO/Ag/Ag_2_WO_4_ heterostructure showed a reasonable response upon illumination by solar light, whereas the response at dark is relatively shows very low and flat curves were observed. However, the *I*–*V* curve of the ZnO/Ag/Ag_2_WO_4_ photo-electrode under simulated sun light confirms a higher photoelectric conversion than that of the ZnO NRs photo-electrode. The observed photocurrent density at the potential of 1.23 V (*vs.* Ag/AgCl) is 0.9 mA cm^−2^ for ZnO NRs and increased by factor of three to 3 mA cm^−2^ for the ZnO/Ag/Ag_2_WO_4_ photo-electrode. This result might be attributed to the higher separation and transportation of photo-induced charge carriers^25^ due to the presence of the Ag/Ag_2_WO_4_ particles that affected the band gap of the heterostructure. In addition to that, the presence of metallic Ag^0^ particles (as discussed above in the XPS analysis) would enhance the absorption of visible light and then improve the separation rate of the photo-generated electrons–holes pairs because of the SPR effect which can locally amplifies the incident electromagnetic field at the metal surface by several orders of magnitude.^12,29^

**Fig. 9:**
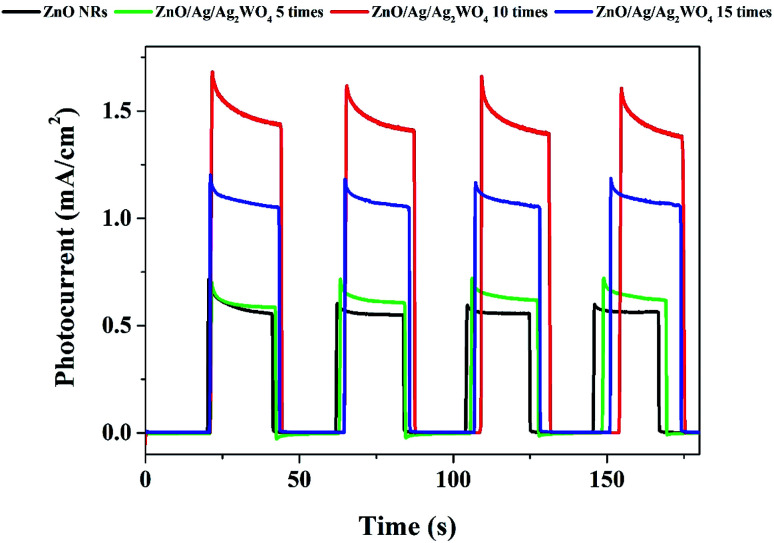
Chronoamperometry *I*–*t* curves of the ZnO NRs, and the ZnO/Ag/Ag_2_WO_4_ photo-electrodes under chopped illumination of solar light with applied voltage of +5 V with 20 s light on/off cycles.

The photo response over time of the samples were investigated through the chronoamperometry measurements which record the photocurrent density *versus* time in dark (light off) and under illumination (light on) with an applied potential of 0.5 V as shown in Fig. 9. From Fig. 8 we could see the result with different amount of Ag/Ag_2_WO_4_ that was prepared by different SILAR cycles. It is clear that the photo response increases with increasing the number of SILAR cycles. However, the photocurrent is decreased when the deposition cycle increased up to 15 times. The possible reason for that is the effect of additional deposition cycles lead to the formation of larger aggregates around the ZnO NRs. In turn, this might cause a destruction of the junctions and the result of that is that the separation of the charge carriers at the interfaces of the heterojunction will be reduced. The photocurrent density of the ZnO was found to be 0.6 mA cm^−2^, and it is increased to 1.6 mA cm^−2^ after deposition of Ag/Ag_2_WO_4_ 10 times on the ZnO NRs.

**Fig. 10:**
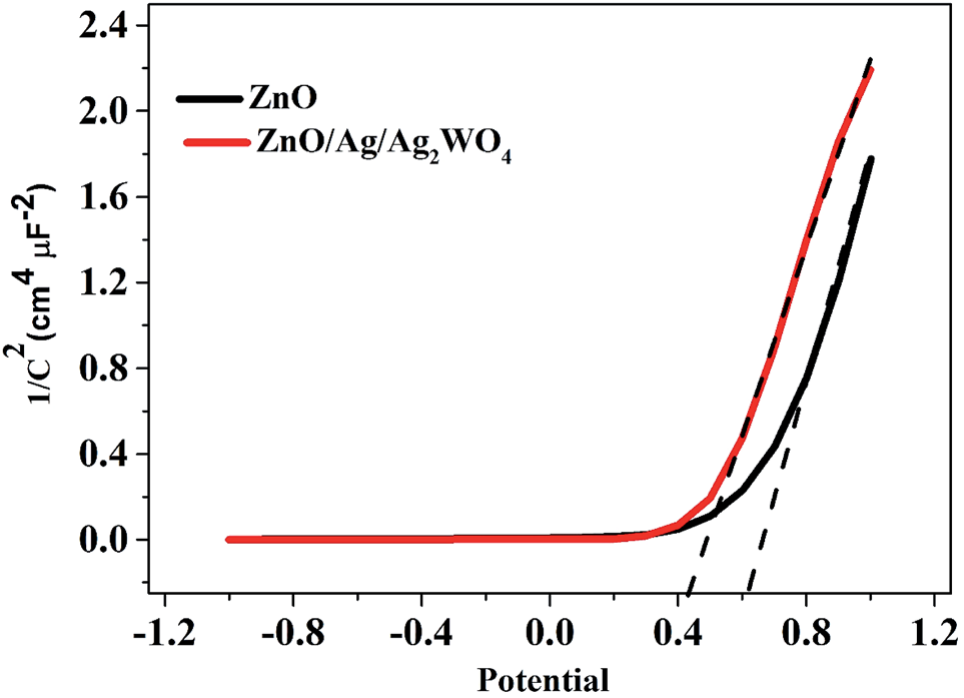
Mott–Schottky plots of 1/*C*^2^*versus* applied potential (V) in complete darkness at a frequency of 3 kHz for the ZnO NRs, and the ZnO/Ag/Ag_2_WO_4_ photo-electrodes.

To understand the electronic properties of the ZnO/Ag/Ag_2_WO_4_ in contact with the electrolyte solution, we performed electrochemical impedance measurement in dark and Mott–Schottky (M–S) plot (1/*C*^2^*versus* potential) was analyzed. One can extrapolate the position of the flat band potential *V*_FB_ (*versus* Ag/AgCl) from the *x*-axis intercept at selected frequency (3 kHz), which was found to be +0.60 and +0.4 V for ZnO and ZnO/Ag/Ag_2_WO_4_ photo-electrodes, respectively (see Fig. 10). The shift in *V*_FB_ is suggesting the presence of more surface states which could lead to considerable change in the band position.^34,35^ The positive slopes were determined from M–S plot indicated the n-type nature of the samples. From the dielectric constant of ZnO (*ε* = 10) and the permittivity of vacuum (*ε*_0_ = 8.85 × 10^−14^ F cm^−1^) the charge carrier density can be calculated from eqn (1)^18,31^1
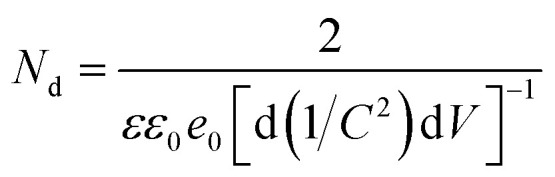


The charge carrier densities were found to be ∼2.8 × 10^19^ and ∼2.5 × 10^19^ cm^−3^ for ZnO NRs and ZnO/Ag/Ag_2_WO_4_ photo-electrodes, respectively which are of the same order. The estimated values of flat band potential and charge carrier densities are in the agreement with those reported previously in the literature.^26^

The incident photon to current conversion efficiency (IPCE) is used in PEC to measure the efficiency of converting an individual photon to an extractable electron. Which performed with a monochromator light source to have a spectral distribution that is selective by wavelength in the range (300–700 nm), and at the same time the current density generated at each wavelength were measured. Then IPCE is calculated from eqn (2):^36,37^2
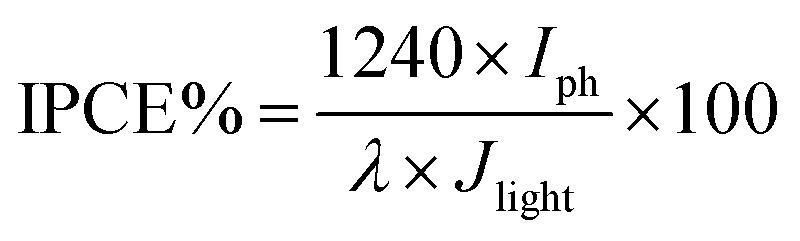
where *I*_ph_ is the photocurrent density, *λ* is the incident wavelength and *J*_light_ is the incident irradiation.

**Fig. 11:**
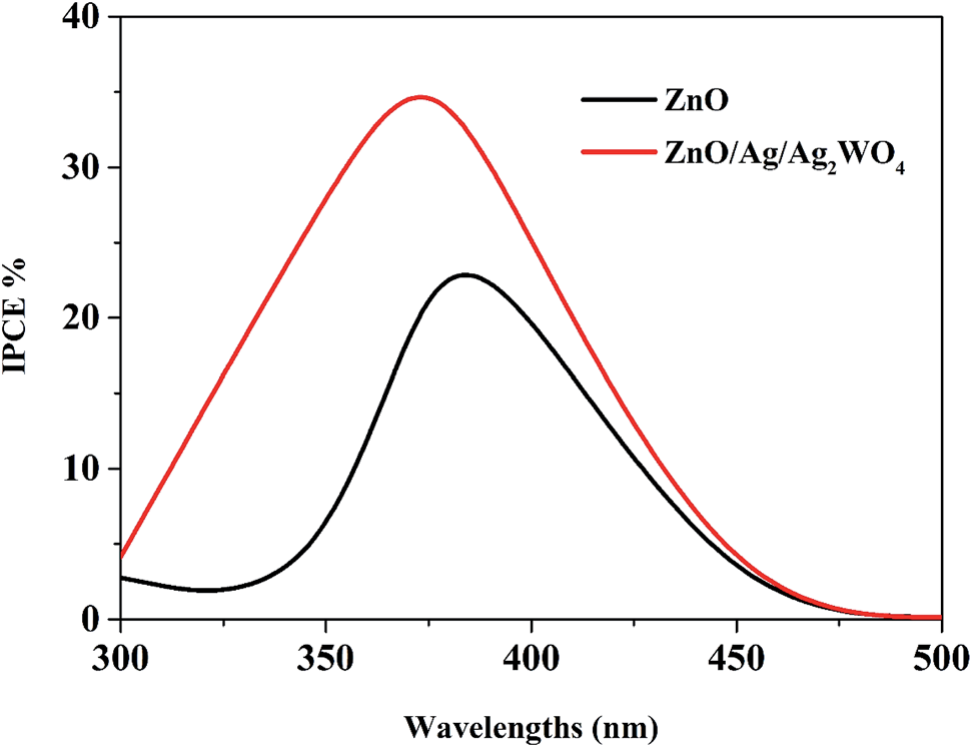
The plots of IPCE *versus* wavelength for the ZnO NRs, and the ZnO/Ag/Ag_2_WO_4_ photo-electrodes.

Fig. 11 compares the IPCE curves for ZnO NRs, and ZnO/Ag/Ag_2_WO_4_ photo-electrodes. In general, the IPCE curves of the pristine ZnO NRs and the ZnO/Ag/Ag_2_WO_4_ photo-electrodes are consistent with the optical absorption spectra of the pristine ZnO NRs, and ZnO/Ag/Ag_2_WO_4_ photo-electrodes. The ZnO photo-electrode exhibits PEC activity on the UV region and exhibits some activities in the visible region but it is relatively weaker than that for the UV region. For the ZnO/Ag/Ag_2_WO_4_ photo-electrode, the photo response range of IPCE is slightly extended in the UV and visible light region in addition to the increase in IPCE, in accordance with the improved optical absorption including increased absorption and extended absorption region of the ZnO/Ag/Ag_2_WO_4_. The extension of the IPCE in the visible region between 400 to 450 is suggested to be due to the effect of SPR. The enhancement in the IPCE is more remarkable than the increase in optical absorption of the ZnO/Ag/Ag_2_WO_4_ as compared to that of the ZnO NRs, which is increased by factor of 1.5. At the wavelength 400 nm the IPCE value is 30% for ZnO/Ag/Ag_2_WO_4_ and 20% for ZnO NRs. This can be attributed to the role of Ag^0^ that is embedded during the sample preparation. The Ag^0^ is expected to facilitate the transfer of electrons generated in ZnO and Ag_2_WO_4_ under solar illumination in the PEC process.^38^

### 3.3 Proposed photoelectrochemical mechanism for water oxidation

It is known to all that the enhancement of PEC activity of semiconductor-based photo-electrodes was mainly attributed to electrons and holes transfer at the interfaces of the photo-electrodes. It is clear that the band edge potential values of the ZnO and the Ag/Ag_2_WO_4_ materials played an important role in the efficiency of generation and separation process of the electrons and holes pairs. The conduction band (CB) and valence band (VB) edge potential of a semiconductor at the point of zero charge can be estimated by the Mulliken electronegativity theory:

3 *E*_VB_ = *X* − *E*^e^ + 0.5*E*_g_

where *E*^e^ is the energy of free electrons on the hydrogen scale (about 4.5 eV) and *E*_VB_ is the VB edge potential. *X* is the absolute electronegativity of the semiconductor, and *E*_g_ is the band gap energy of the semiconductor. Meanwhile, the CB edge potential (*E*_CB_) can be calculated by the equation:

4 *E*_CB_ = *E*_VB_ − *E*_g_

**Fig. 12:**
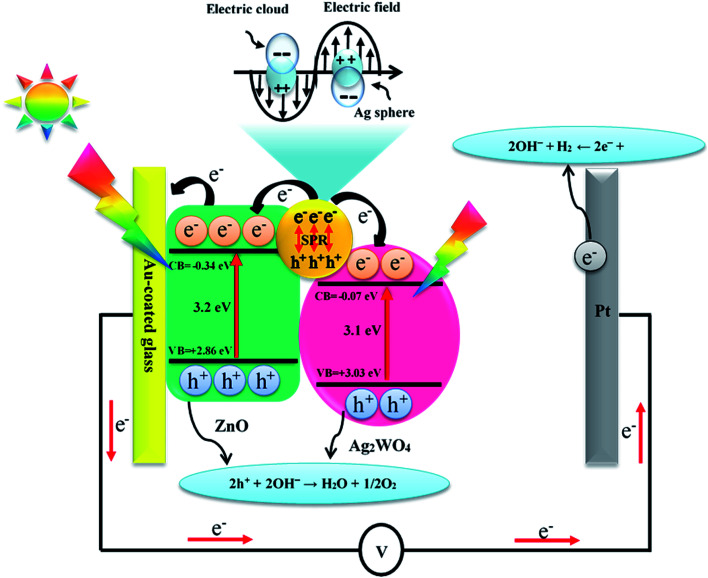
Schematic diagram showing the energy band structure and possible electron–hole separation and transportation in ZnO/Ag/Ag_2_WO_4_ heterostructure with SPR effect.

Here, the *X* values for ZnO and Ag_2_WO_4_ are about 5.76 and 5.98 eV, and the *E*_VB_ of ZnO and Ag_2_WO_4_ are calculated to be +2.86 and +3.03 eV, respectively.^20^ Moreover, *E*_CB_ of ZnO and Ag_2_WO_4_ are estimated to be −0.34 and −0.07 eV, respectively. Depending on the above results, a probable mechanism of the PEC activity can be described as illustrated in Fig. 12. In the presence of solar light, both the semiconductors absorb light and the electrons in the VB get excited up to a higher potential of −0.07 eV for the Ag_2_WO_4_ and −0.34 eV for the ZnO. Therefore, the effective charge transfer process proceeds within the semiconductor due to high photon energy. Due to the SPR effect, Ag^0^ nanoparticles causes effective separation of electron/hole pairs upon absorption of visible light. Electrons from the Ag nanoparticles are transferred to the CB of the ZnO and the Ag_2_WO_4_, while holes remain in the Ag nanoparticles. Meanwhile, the photogenerated electrons in the CB of C will be transferred to the Ag nanoparticles to occupy the vacant holes generated by the plasmonic absorption.^20,39^ With this mechanism, the photo-generated charge carriers can be efficiently separated, resulting in an enhanced PEC performance. Furthermore, the photo-generated electrons will ultimately arrive at the Pt counter electrode and contribute to H_2_ production. Also, the photo-generated holes in the valence band of ZnO NRs and Ag_2_WO_4_ will contribute on O_2_ generation through water oxidation. Therefore, these results confirm that the Ag/Ag_2_WO_4_ modification is an effective way to obtain a high PEC activity using ZnO NRs arrays.

## 4 Conclusion

In summary, we report a preparation of ZnO/Ag/Ag_2_WO_4_ photo-electrode for PEC water oxidation with surface plasmonic resonance behavior *via* low temperature hydrothermal chemical growth flowed by SILAR method. The structural and morphology characterization studies revealed that ZnO/Ag/Ag_2_WO_4_ heterostructure was prepared successfully. Whereas the EDX and XPS characterization confirms the elemental composition of ZnO/Ag/Ag_2_WO_4_ which consist of Zn, O, Ag, and W without any other elements detected, and the energy state of the elements on ZnO/Ag/Ag_2_WO_4_ heterostructure. ZnO/Ag/Ag_2_WO_4_ heterostructure shows an obvious red shift of the optical absorption in the visible region than that of pristine ZnO NRs with calculated optical band gaps of 3.2, and 3.1 eV for ZnO NRs and ZnO/Ag/Ag_2_WO_4_, respectively. Compared with ZnO NRs, the ZnO/Ag/Ag_2_WO_4_ exhibits a higher PEC performance. By the deposition of the Ag/Ag_2_WO_4_ on the ZnO NRs, a new heterostructure was obtained *via* SILAR method, leading to higher photocurrent of 3 mA cm^−2^ measured at 1.23 V (*vs.* Ag/AgCl) for ZnO/Ag/Ag_2_WO_4_, which is 3 times the photocurrent achieved by ZnO NRs photo-electrode. Also, the photo response over time shows a higher photocurrent for the ZnO/Ag/Ag_2_WO_4_ photo-electrode (1.6 mA cm^−2^) compared to that of the ZnO NRs photo-electrode (0.6 mA cm^−2^). In addition to that, the overall IPCE of the ZnO/Ag/Ag_2_WO_4_ photo-electrode was observed to increase by a factor of 1.5 compared to the ZnO NRs photo-electrode with extension of the IPCE curve in the visible light region due to the SPR effect. The high PEC performance of our samples could be attributed to the higher separation and transportation of photo-induced charge carriers. This is due to the enhancement in the absorption of visible light which improves the separation rate of the photo-generated electrons–holes pairs because of the band gap engineering and to the SPR effect of the metallic silver that was introduced during the sample preparation. Our study exposes the potential of ZnO/Ag/Ag_2_WO_4_ photo-electrode for high performance in PEC water splitting.

## Conflicts of interest

There are no conflicts of interest to declare.

## Supplementary Material
